# Hydrogen sulfide and ferroptosis inhibition underlies the dietary restriction-induced protection against cyclophosphamide cystitis

**DOI:** 10.3389/fphar.2025.1562852

**Published:** 2025-05-13

**Authors:** Zhimin Mao, Ping Chen, Qun Ji, Xiuling Zhao, Kun Zhong, Xuhui Zeng

**Affiliations:** Institute of Reproductive Medicine, Medical School, Nantong University, Nantong, Jiangsu, China

**Keywords:** dietary restriction, hydrogen sulfide, cyclophosphamide, acrolein, cystitis, oxidative stress, ferroptosis

## Abstract

Dietary restriction (DR) has emerged as a potential therapeutic intervention for various pathological conditions. This study investigated the effects of DR on cyclophosphamide-induced cystitis in mice. Animals were subjected to controlled food restriction for 1 week prior to cyclophosphamide administration. We evaluated changes in body weight, bladder pathology, redox status, and ferroptotic parameters. DR significantly attenuated cyclophosphamide-induced cystitis severity, as evidenced by reduced bladder weight, decreased lipid peroxidation, and diminished ferroptotic markers in bladder tissue. Mechanistic investigations revealed that DR upregulated hepatic hydrogen sulfide (H_2_S)-synthesizing enzymes and enhanced H_2_S production. Inhibition of H_2_S-synthesizing enzymes with DL-propargylglycine (PAG) and aminooxyacetic acid (AOAA) exacerbated cyclophosphamide-induced cystitis, whereas administration of diallyl trisulfide (DATS), an H_2_S donor, markedly ameliorated bladder pathology. *In vitro* studies demonstrated that H_2_S donors, NaHS and DATS, protected against cyclophosphamide metabolite acrolein (ACR)-induced urothelial cell death by suppressing oxidative stress, as indicated by reduced p38 MAPK activation and protein carbonylation. These findings suggest that DR confers protection against cyclophosphamide-induced cystitis through the induction of endogenous H_2_S production and inhibition of ferroptosis. Our study provides additional evidence supporting the health-promoting effects of DR as well as novel mechanistic insights into the beneficial effects of DR. Given H_2_S has anti-inflammatory and anti-oxidative properties and that oxidative stress and ferroptosis underlie various diseases, our finding could have broader implications.

## 1 Introduction

Cyclophosphamide (CYP) is a widely used chemotherapeutic agent that, despite its therapeutic efficacy, can cause severe adverse effects, including hemorrhagic cystitis. This debilitating condition affects many patients receiving CYP therapy and significantly impacts their quality of life (Ruggeri et al., 2015; Gutiérrez-Aguirre et al., 2022). The pathogenesis of CYP-induced cystitis primarily involves the generation of acrolein (ACR), a toxic metabolite that triggers oxidative stress, inflammation, and cellular damage in the bladder epithelium (Cox, 1979; Korkmaz et al., 2007).

Recent studies from our group and others have shown that oxidative stress and ferroptosis, a form of regulated cell death characterized by iron-dependent lipid peroxidation, play crucial roles in the development of CYP-induced cystitis (Korkmaz et al., 2007; Mao et al., 2023a; Zhang et al., 2006). The accumulation of lipid peroxidation products and the dysregulation of cellular antioxidant systems, particularly the glutathione (GSH)-dependent pathways, have been implicated in the pathogenesis of bladder inflammation and injury. Although several effective drugs and strategies are available to minimize or treat hemorrhagic cystitis caused by CYP, there remains a need to explore novel, simple, and natural approaches for its prevention and treatment.

Dietary restriction (DR) is defined as a reduction in overall food intake without causing malnutrition, which has emerged as a promising intervention for various pathological conditions. While the specific caloric reduction required to achieve health benefits can vary depending on individual factors, DR generally involves a reduction of 20%–40% in caloric intake compared to *ad libitum* (unrestricted) feeding (Lee and Longo, 2016). Numerous studies have demonstrated that DR can extend lifespan, improve metabolic health, and enhance stress resistance across multiple species (Souza Matos et al., 2021; Castro-Barquero et al., 2020; Chao et al., 2021; Fontana et al., 2010). The beneficial effects of DR are attributed to various mechanisms, including reduced oxidative stress, enhanced autophagy, and improved cellular repair mechanisms (Walsh et al., 2014; Bagherniya et al., 2018; Jafari et al., 2024). However, the potential protective effects of DR against chemical-induced bladder injury and the underlying mechanisms remain unexplored.

Hydrogen sulfide (H_2_S), a gaseous signaling molecule, has gained significant attention for its crucial role in cellular homeostasis and stress response. Endogenous H_2_S is primarily synthesized by three enzymes: cystathionine β-synthase (CBS), cystathionine γ-lyase (CSE), and 3-mercaptopyruvate sulfurtransferase (3-MST). Growing evidence suggests that H_2_S exhibits potent antioxidant properties and can protect against various forms of cellular injury (Zhang et al., 2019; Liu et al., 2024; Wang et al., 2022). The H_2_S system has been shown to modulate multiple cellular processes, including redox balance, mitochondrial function, and cell death pathways (Kar et al., 2019; Murphy et al., 2019; Xie et al., 2016). Notably, recent studies have indicated that H_2_S levels can be influenced by dietary interventions, suggesting a potential link between nutritional status and H_2_S-mediated protective mechanisms (Hine et al., 2018; Bithi et al., 2021; Hine et al., 2015). Recently, we have characterized H_2_S as an important protective mechanism against ACR-induced cell and tissue injuries (Mao et al., 2023b; Mao et al., 2022). There are also studies showing the protective effects of H_2_S against various forms of organ injury (Ni et al., 2021; Zhang H. et al., 2023; Cui et al., 2016). However, it is currently unclear whether DR protects against chemical-induced bladder injury. The purpose of this study was to address this possibility.

In this study, we investigated the potential protective effects of DR against CYP-induced cystitis and explored the underlying mechanisms, with a particular focus on the H_2_S signaling pathway. Here, we present our results showing the short-term DR significantly enhanced body defense against CYP-induced and ACR-mediated cystitis via mechanisms involving induction of endogenous H_2_S generation and suppression of oxidative stress and ferroptotic bladder cell injury. Our findings provide novel insights into the mechanisms by which DR influences tissue resistance to chemical injury and highlight the potential therapeutic implications of targeting the H_2_S pathway in bladder inflammatory conditions.

## 2 Materials and methods

### 2.1 Materials

Cyclophosphamide (CYP, #PHR1404), sodium hydrosulfide hydrate (NaHS, #161527) and DL-Propargylglycine (PAG, #P7888) were purchased from Sigma-Aldrich (St Louis, MO, United States). Aminooxyacetic acid hemihydrochloride (AOAA, #HY-107994) was from MedChemExpress (Shanghai, China). Diallyl trisulfide (DATS, #D854967) was obtained from Macklin (Shanghai, China). Acrolein (ACR, #ZD816) was purchased from Xianding Biotechnology (Shanghai, China). Antibodies against γ-H2A.X (#9718), GPX4 (#52455), phospho-p38 mitogen activated protein kinase (Thr180/Tyr182) (p-P38MAPK, #8203S), α-tubulin (#3873T), rabbit IgG (#5366), and mouse IgG (#5470) were from Cell Signaling Technology (Danvers, MA, United States). Anti-SCL7A11 antibody (#26864-1-AP) was from Proteintech (Chicago, IL, United States). Antibodies against ACSL4 (#ab155282) and ACR (#ab240918) were from Abcam (Cambridge, United Kingdom). Anti-CTH (#sc-374249) and CBS (#sc-133154) antibodies were obtained from Santa Cruz Biotechnology (Santa Cruz, CA, United States). Anti-GAPDH (#GB11002) and β-actin (#GB12001) antibodies were from Servicebio Technology (Wuhan, China).

### 2.2 Mice and mice dietary restriction

For all experiments, adult male ICR mice aged 8 weeks were used. The mice were housed in the animal facilities and provided free access of food and water in a temperature-controlled room with a 12 h light/dark cycle. All animal experimental procedures were performed in accordance with the animal care and use committee of Nantong University and approved under protocol number S20221222-005.

DR was implemented following methods previously described by Bithi et al. (2021), Hine et al. (2015). Briefly, the average daily food intake of ad libitum-fed mice was monitored over a period to establish a baseline. Afterward, the mice were subjected to DR by receiving 50% of their baseline food intake for 7 days. At the conclusion of this DR period, the mice were used for the induction of CYP-induced cystitis.

### 2.3 Cells

SV-HUC-1 cells (#CL-0222) were bought from Procell Life Science & Technology (Wuhan, China). The cells were cultured in Dulbecco’s Modified Eagle Medium (DMEM)/F-12 (#11320-033, Invitrogen) supplemented with 10% fetal bovine serum (FBS, #F8687, Sigma-Aldrich) for routine maintenance and expansion. The cells were cultured in DMEM/F-12 containing 1% FBS for experimental purposes.

### 2.4 Western blot analysis

Western blot analysis was performed as described previously (Mao et al., 2022). Briefly, equal amounts of bladder tissue samples or cell protein samples were separated by 8%–20% SDS-PAGE and transferred onto polyvinylidene fluoride (PVDF) membranes (#IPVH00010, Merck Millipore). The membranes were blocked with 5% non-fat dry milk for 1 h to prevent the no-specific antibody binding. This was followed by a reaction with the corresponding primary or secondary antibodies at 4°C for 2 h. The signal in protein bands was captured and visualized using the Amersham Imager 600 (GE Healthcare). Equal protein loading in each lane was confirmed with anti-GAPDH, anti-β-actin, anti-α-tubulin antibodies or EZBlue Gel Staining (#G1041, Sigma-Aldrich). Quantification of bands was performed using ImageJ software.

### 2.5 Protein oxidation assessment

Protein carbonylation was detected using the OxyBlot Protein Oxidation Detection Kit (#S7150, EMD Millipore), as reported previously (Mao et al., 2022). Briefly, protein lysates were prepared by suspending cell proteins in RIPA buffer. Five microliters of protein samples containing 15–20 µg of protein were mixed with 5 µL of 12% SDS and 10 µL of DNPH (2,4-dinitrophenyl hydrazine) solution to denature and derivatize the proteins, respectively. After a 15-min incubation at room temperature, 7.5 µL of neutralization solution was added, and the samples were subjected to Western blot analysis. Equal protein loading in each lane was confirmed using EZBlue Gel Staining. Protein carbonylation levels were quantified using ImageJ software.

### 2.6 Malondialdehyde (MDA) detection

The MDA level in mouse bladder was measured with thibabituric acid (TBA) method using a commercial kit from the Nanjing Jiancheng Bioengineering Institute (Nanjing, China), following the protocol provided by the manufacturer.

### 2.7 Determination of H_2_S-producing capacity

H_2_S-producing capacity was assayed as previously described by Hine and Mitchell (2017). The H_2_S test paper was made by soaking the filter paper in 20 mM lead acetate solution and dried. For determination of H_2_S-producing capacity, tissue lysates or cells were placed into a 96-well plate in DMEM/F12 containing 10 mM L-cysteine and 10 μM pyridoxal-5′-phosphate and the H_2_S test paper was placed directly over the plate for the indicated times. The formation of the visible black-colored circle on the paper, resulting from the reaction of the released gaseous H_2_S and lead acetate, was photographed and analyzed with ImageJ software.

### 2.8 Haematoxylin and eosin (H&E) staining

Bladder tissues collected from the mice were fixed with 4% paraformaldehyde at room temperature for 72 h and embedded in molten paraffin. Five-micrometer-thick sections were cut using a microtome and subjected to H&E staining for tissue histology. The H&E staining was conducted using standard procedure. Briefly, the sections were initially deparaffinized in xylene and rehydrated through an ethanol gradient. Nuclei were stained with haematoxylin for 2 min, followed by differentiation in 1% acid ethanol for 3 s and rinsing in distilled water. Then cytoplasm was stained with eosin solution for 2 min. Finally, sections were dehydrated in an ethanol gradient and cleared in xylene. To evaluate bladder pathology, tissue samples from each experimental group (n = 2 mice, 5 replicates per group) were systematically examined under light microscopy.

### 2.9 Maleimide-labeling assay

Bladder proteins were extracted and allowed to react with 2.5 μM Alexa Fluor 680 C2-maleimide (#A20344, Thermo Scientific) at 4°C for 2 h. Afterward, samples were separated by 12% SDS-PAGE, and the fluorescent signal in the gel was captured using Amersham Imager 600. The equal loading of proteins was confirmed by EZBlue Gel Staining.

### 2.10 Calcein-AM/propidium iodide (PI) staining

Cells in 96-well plates were incubated with a mixture of Calcein-AM and PI (#C542, Dojindo) for about 10–20 min. Viable cells were stained green with Calcein-AM due to intracellular esterase activity, while dead cells with compromised plasma membranes were stained red with PI. Stained cells were visualized and imaged under a fluorescence microscope.

### 2.11 WST assay

Cells cultured in 96-well plates were incubated with WST (#CK04, Dojindo) for approximately 30 min, and the absorbance at 450 nm was measured using a spectrometer.

### 2.12 Statistical analysis

The data are presented as mean ± standard error (S.E.). Student’s *t*-test was used for the comparison of the two groups. One-way ANOVA analysis and Post-hoc Dunnett comparisons were performed for multiple comparisons. The analyses were done either with Microsoft Excel (Microsoft, Redmond, WA, United States) or Sigmaplot software (Systat Software Inc., San Jose, CA, United States). *P* < 0.05 was considered statistically significant.

## 3 Results

### 3.1 DR attenuates CYP-induced cystitis and ferroptotic bladder injury

**FIGURE 1:**
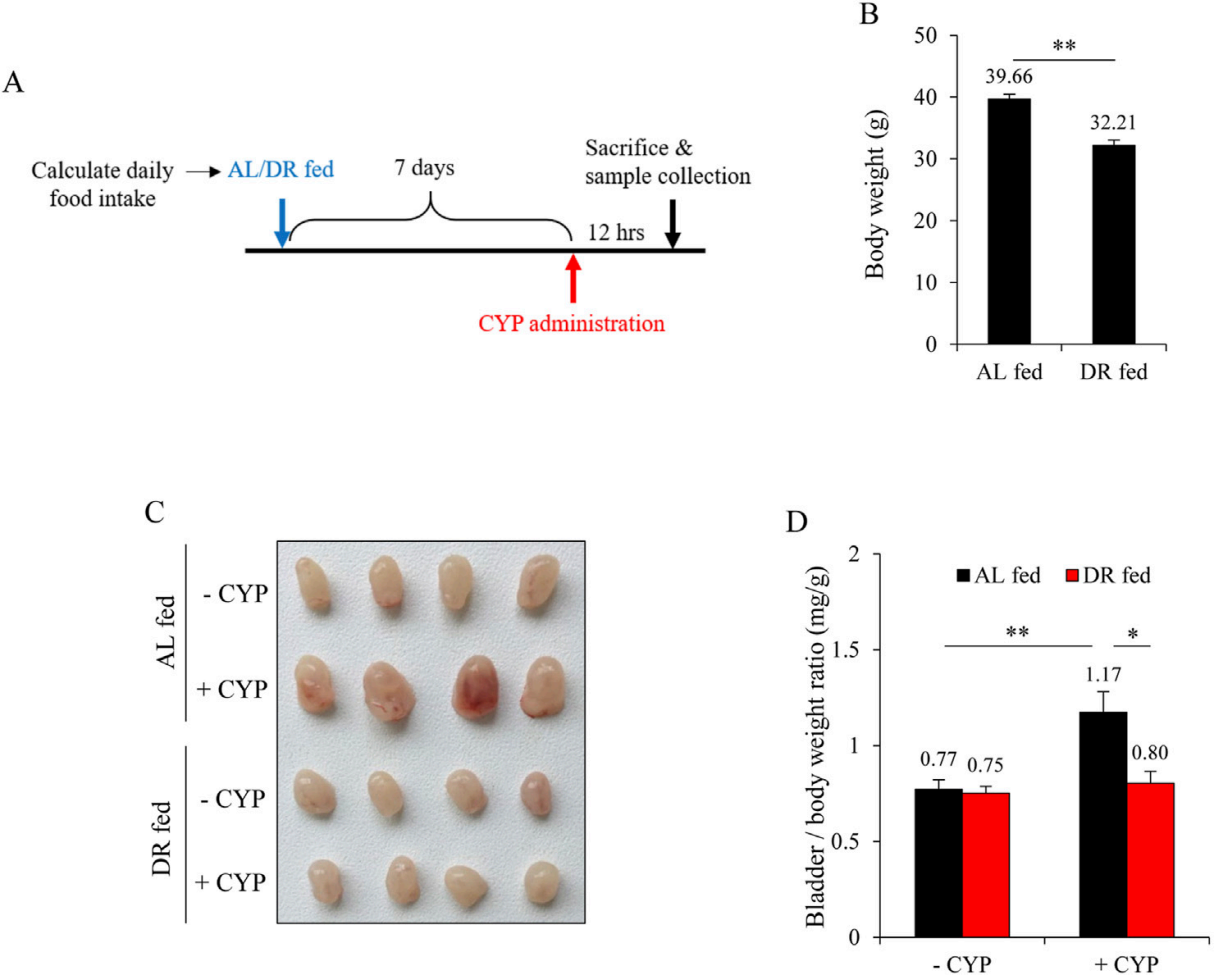
Dietary restriction (DR) mitigates cyclophosphamide (CYP)-induced cystitis. **(A)** Schematic of the experimental design. Mice were monitored for daily food intake over a period, followed by either *ad libitum* (AL) feeding or a 50% reduction in their normal diet (DR) for 7 days. Subsequently, mice were given an intraperitoneal injection of 150 mg/kg CYP, and bladder changes were assessed 12 h later. **(B)** Effect of DR on body weight prior to CYP intervention. Data shown are mean ± S.E. (n = 10; **P < 0.01). **(C)** Effect of DR on CYP-induced cystitis. The AL fed-CYP group showed significant congestion, enlargement, and edema, while the DR fed-CYP group exhibited marked improvement. **(D)** Bladder weight-to-body weight ratio in AL fed and DR fed mice with or without CYP treatment. Data shown are mean ± S.E. (n = 4; *P < 0.05, **P < 0.01).

To investigate the effects of DR on CYP-induced bladder injury, we implemented a controlled feeding protocol as described by Bithi et al. (2021), Hine et al. (2015). Initially, the daily food intake of the mice was measured to establish a baseline. Subsequently, the mice were subjected to DR by being provided with 50% of their normal food intake for 7 days. Following this period of DR, the mice were then induced with CYP cystitis to assess the resulting changes in bladder injury compared to a control group (Figure 1A). Figure 1B shows that, as compared to *ad libitum* (AL) fed controls, the body weight of DR mice was significantly reduced. CYP administration caused obvious bleeding and edema in AL group, but not in DR group. There were no noticeable macroscopic changes in the bladder (Figure 1C). The increased ratio of bladder to body weight was also significantly reduced in the DR group (Figure 1D).

**FIGURE 2:**
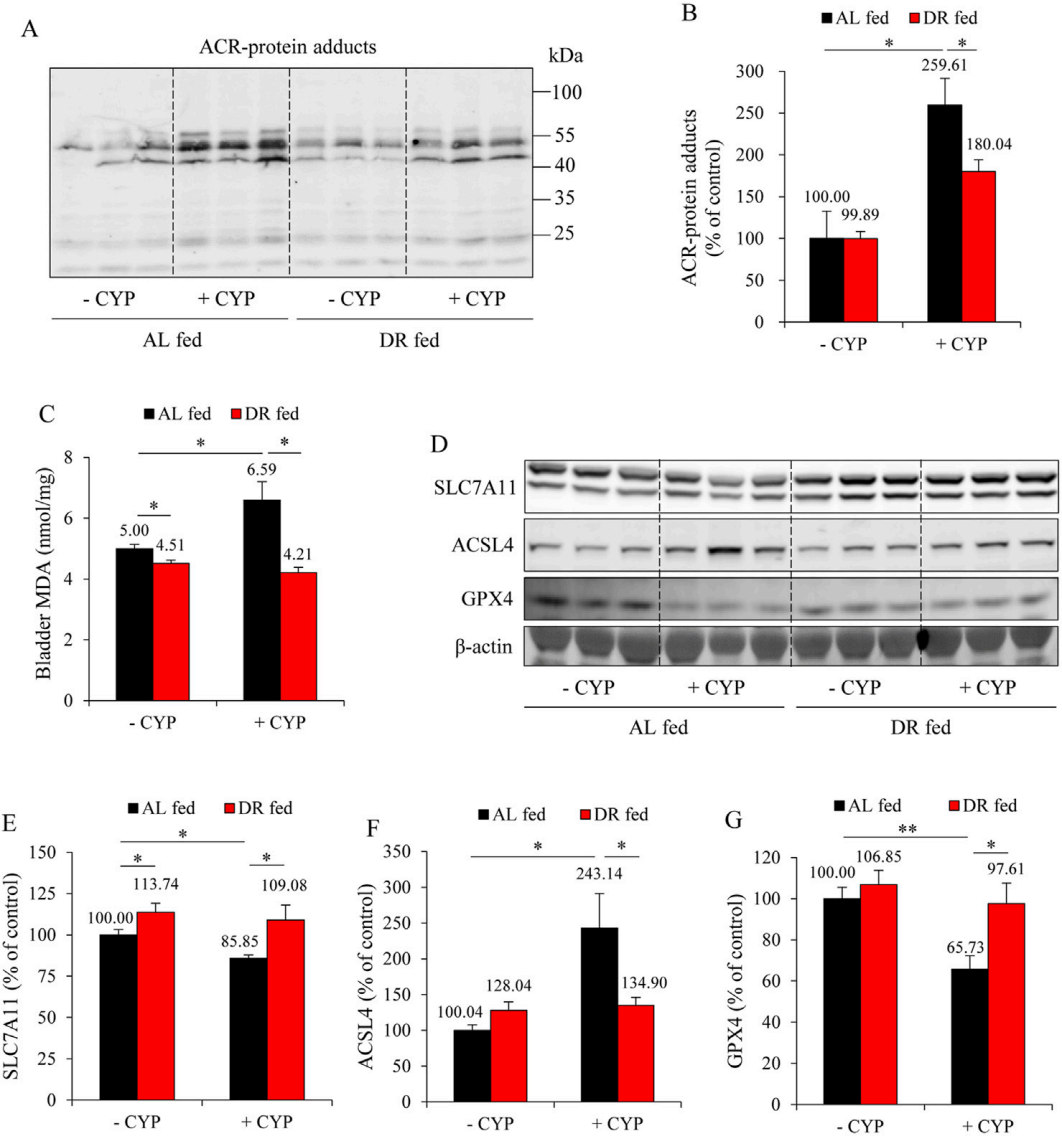
DR prevents ferroptosis in the bladder. **(A,B)** Effect of DR on acrolein (ACR)-protein adducts formation. Bladder proteins were subjected to Western blot for ACR adducts **(A)**. Densitometric analysis of the blots was shown in **(B)** and expressed as the percentage of control (mean ± S.E.; n = 3; *P < 0.05). **(C)** Effect of DR on bladder malondialdehyde (MDA) production. The extracted bladder proteins were subjected to MDA assay following the manufacturer’s protocol (mean ± S.E.; n = 3; *P < 0.05). **(D–G)** Effect of DR on ferroptosis markers in mouse bladder. Bladder proteins were analyzed with Western blot for solute carrier family 7a member 11 (SLC7A11), acyl-CoA synthetase long-chain family member 4 (ACSL4), glutathione peroxidase 4 (GPX4) and GAPDH expression, respectively **(D)**. Densitometric analysis of the blots was shown in **(E–G)** and expressed as the percentage of control (mean ± S.E.; n = 3; *P < 0.05, **P < 0.01).

We have previously reported that ferroptosis underlies CYP-induced bladder injury (Mao et al., 2023a). We, therefore, detected the changes in oxidative markers and ferroptotic markers. As shown in Figures 2A,B, CYP administration caused a significant elevation in the formation of ACR adducts in the bladder. It also elevated lipid peroxidation product MDA (Figure 2C). Further analysis revealed that CYP decreased SLC7A11 and GPX4 while it increased ACSL4, indicative of induction of ferroptosis. All these changes, however, were significantly alleviated in the DR group. These results suggest that DR protected mice from CYP-induced bladder ferroptosis (Figures 2D–G).

### 3.2 DR stimulates H_2_S production

**FIGURE 3:**
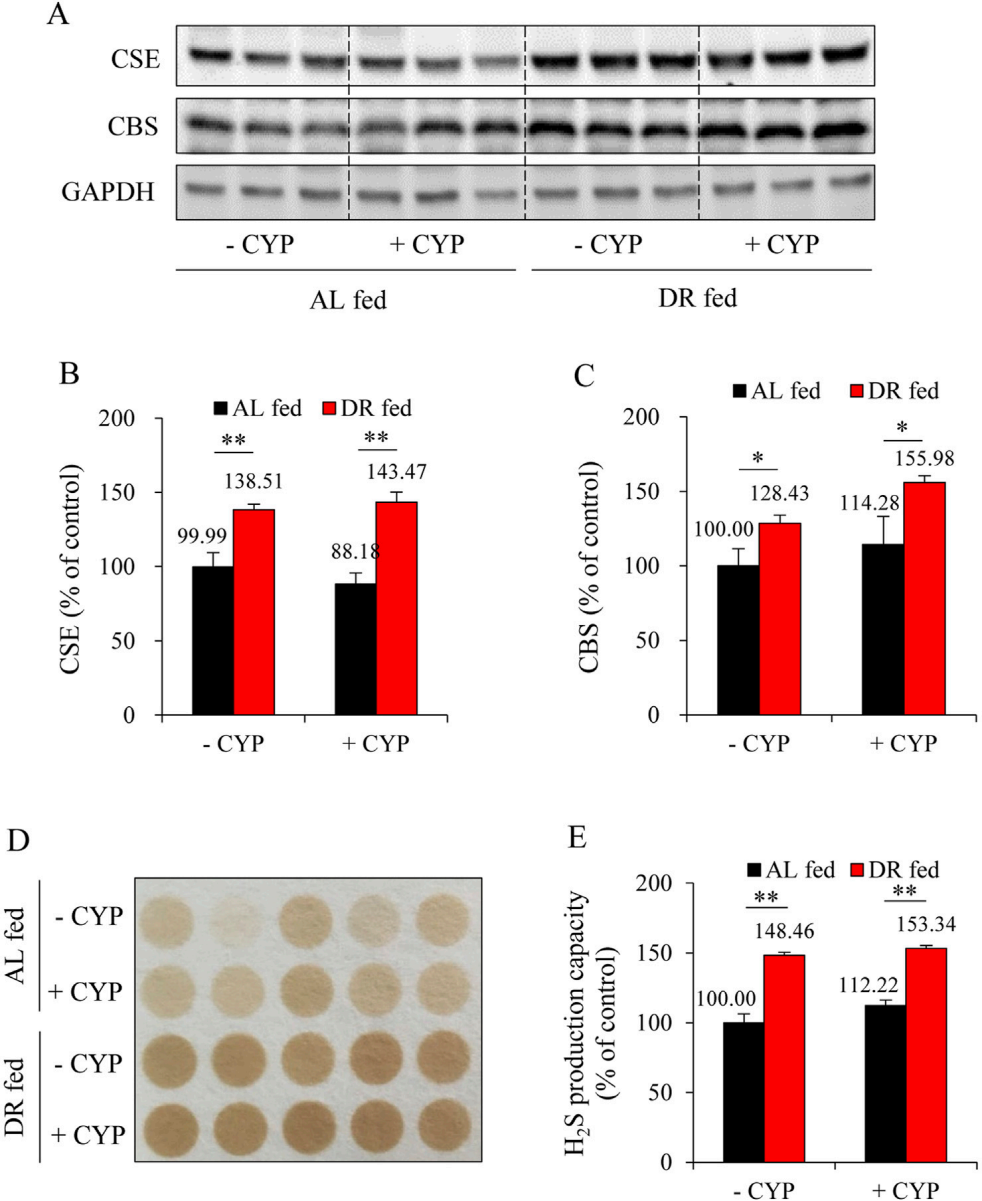
DR enhances hydrogen sulfide (H_2_S) production in the liver. **(A–C)** Effect of DR on cystathionine γ-lyase (CSE) and cystathionine β-synthase (CBS) expression in liver. Liver proteins were extracted and subjected to Western blot for H_2_S-synthesizing enzymes CSE and CBS **(A)**. Densitometric analysis of the blots was shown in **(B,C)** and expressed as the percentage of control (mean ± S.E.; n = 3; *P < 0.05, **P < 0.01). **(D,E)** Effect of DR on H_2_S production capacity in liver tissue. Liver proteins were subjected to lead sulfide assay, and the color formed at 2 h after incubation was photographed **(D)**. Densitometric quantification of the intensity of the colored circles was performed, and data shown in **(E)** have been normalized to control (mean ± S.E.; n = 5; **P < 0.01).

Recently, we have characterized H_2_S as a novel scavenger of ACR (Mao et al., 2022). Therefore, we investigated whether DR’s protective action could be due to the increased H_2_S generation. For this purpose, we have focused on the liver because it is the primary source of H_2_S *in vivo* (Kamoun, 2004; Norris et al., 2011), and it is also the organ responsible for ACR production after CYP administration (Marinello et al., 1984). Figures 3A–C show that DR markedly increased expression of H_2_S-synthesizing enzymes, CSE and CBS, in the liver. Consistently, DR mice exhibited a significantly enhanced level of H_2_S-generating potency (Figures 3D,E). These results indicate that DR induces liver H_2_S production.

### 3.3 H_2_S mediates DR-induced bladder protection

**FIGURE 4:**
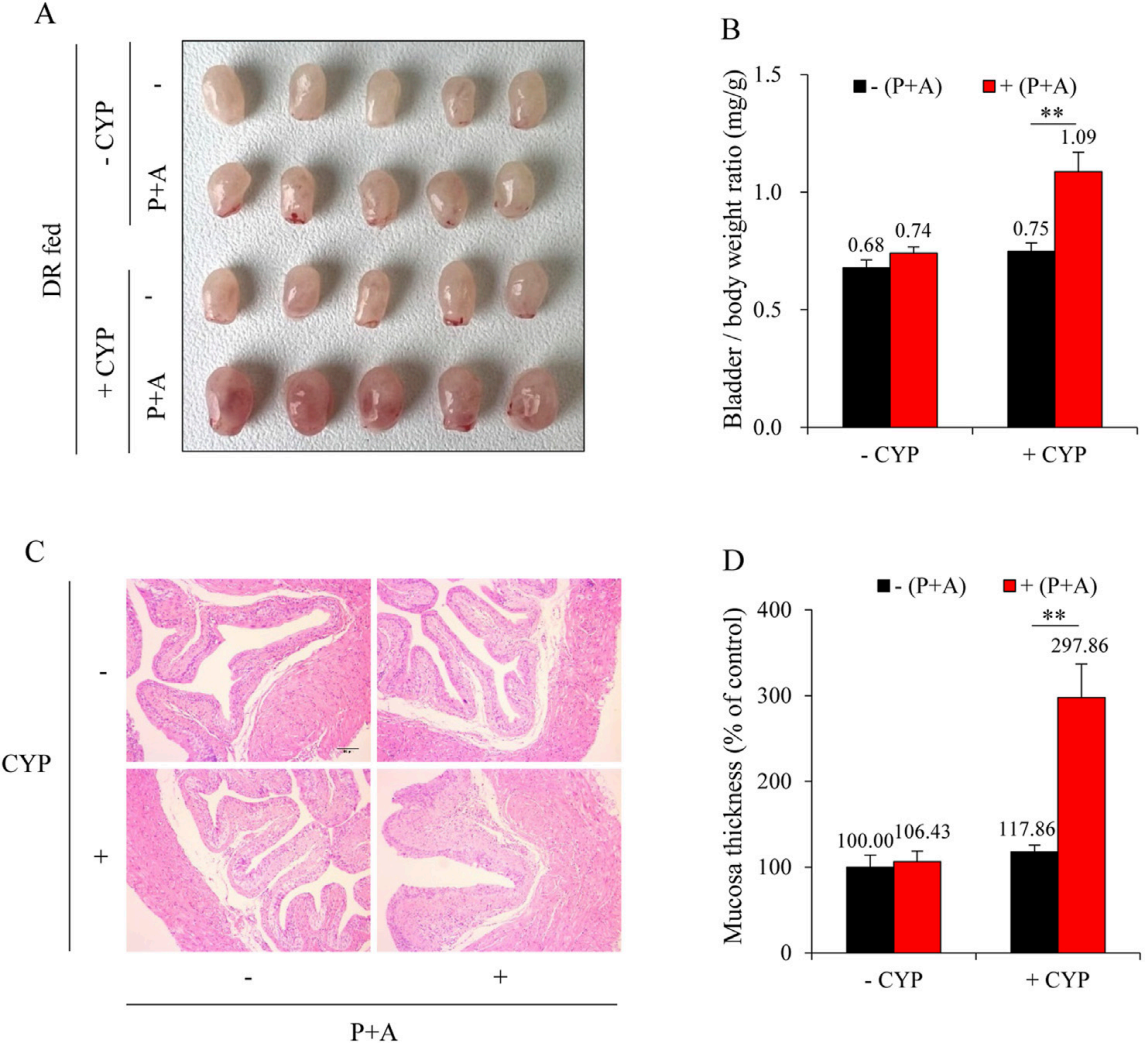
Inhibition of H_2_S synthesizing enzymes reverses the protective effect of DR on CYP-induced cystitis. **(A)** Effect of H_2_S inhibition on bladder morphology. All mice were given DR for 7 days. During the last 36 h, some mice received intraperitoneal injections of a mixture of DL-propargylglycine (PAG, 25 mg/kg) and aminooxyacetic acid (AOAA, 12.5 mg/kg) every 12 h for three times. Afterward, these mice were injected with 150 mg/kg CYP and sacrificed for bladder collection at 12 h. Bladders from DR-fed CYP mice pretreated with PAG and AOAA exhibited significant congestion, enlargement, and edema. **(B)** Effect of H_2_S inhibition on bladder weight-to-body weight ratio. Data are presented as mean ± S.E. (n = 5; **P < 0.01). **(C,D)** Effect of H_2_S inhibition on bladder histopathology. Representative histological images of bladder sections from different treatment groups are shown **(C)**. Scale bar = 100 μm. The thickness of bladder mucosa was analyzed as a percentage of the control **(D)** (mean ± S.E.; n = 5; **P < 0.01).

To elucidate the role of H_2_S in DR-mediated bladder protection, we inhibited the major H_2_S-synthesizing enzymes, CSE and CBS, using the inhibitors PAG and AOAA in DR mice. As shown in Figure 4A, the administration of PAG and AOAA in combination alone did not significantly alter bladder morphology; however, it completely abrogated the protective effects of DR against CYP-induced bladder cystitis. Mice treated with PAG and AOAA exhibited noticeable pathological changes, including pronounced bladder edema, hemorrhage, and increased bladder weight (Figures 4B,C), which were corroborated by histopathological analysis revealing a significant reduction in bladder mucosal thickness (Figure 4D).

**FIGURE 5:**
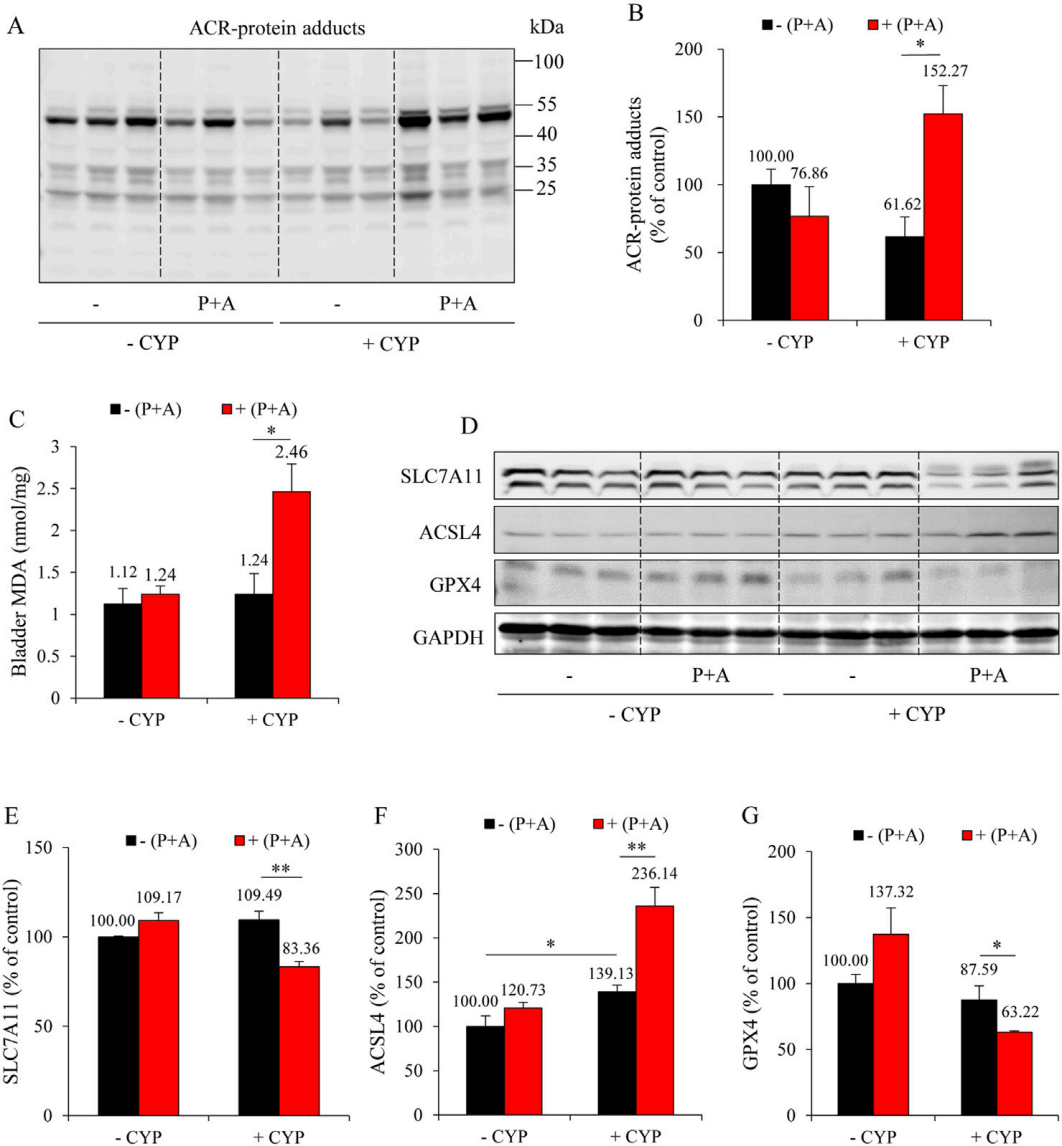
H_2_S inhibition reverses the anti-ferroptotic effect of DR on CYP-induced cystitis. **(A,B)** Effect of H_2_S inhibition on ACR-protein adduct formation. Bladder proteins were subjected to Western blot for ACR adducts **(A)**. Densitometric analysis of the blots was shown in **(B)** and expressed as the percentage of control (mean ± S.E.; n = 3; *P < 0.05). **(C)** Effect of H_2_S inhibition on bladder MDA production. The extracted bladder proteins were subjected to MDA assay following the manufacturer’s protocol (mean ± S.E.; n = 3–5; *P < 0.05). **(D–G)** Effect of H_2_S inhibition on ferroptosis markers in mouse bladder.

Consistent with the macroscopic and histological observations, inhibition of H_2_S synthesis abolished the beneficial effects of DR on ACR-induced oxidative stress and ferroptosis in the bladder. It significantly enhanced lipid peroxidation, as evidenced by the increased formation of ACR-protein adducts (Figures 5A,B) and MDA level (Figure 5C). It also exaggerated the ferroptotic changes, as indicated by the reduced levels of SLC7A11 and GPX4, as well as the increased level of ACSL4 (Figures 5D–G).

**FIGURE 6:**
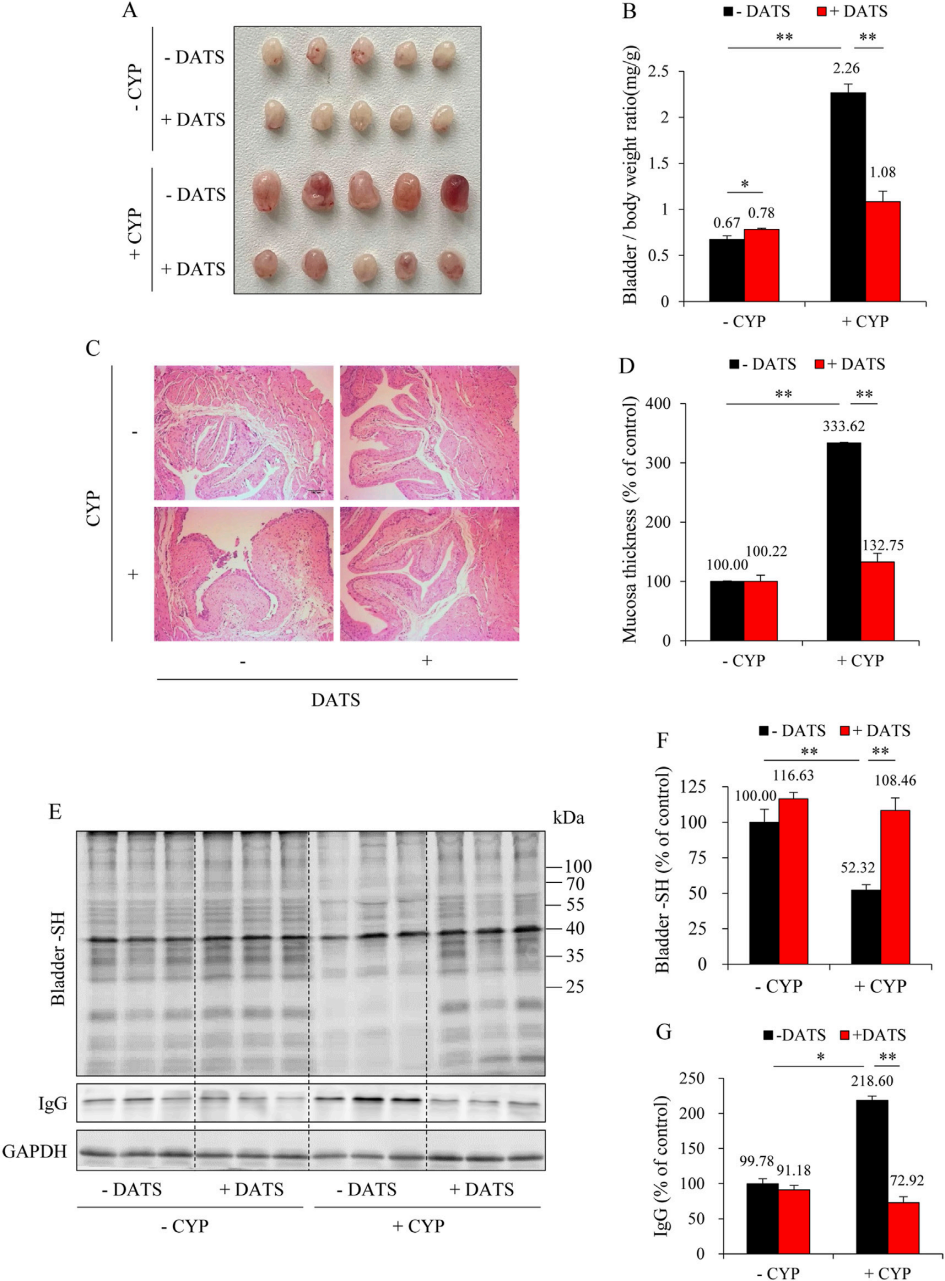
Diallyl trisulfide (DATS) supplementation protects against CYP-induced cystitis. **(A)** Effect of DATS on bladder morphology. Mice were given 60 mg/kg DATS for 3 days via gavage. Afterward, the mice were *i.p.* injected with 150 mg/kg CYP. The bladder changes at 12 h were examined. Note the obvious congestion, enlargement, and edema in bladders in CYP group, but a significant improvement in CYP-DATS group. **(B)** Effect of DATS on bladder weight-to-body weight ratio. Data shown are mean ± S.E. (n = 5; *P < 0.05, **P < 0.01). **(C,D)** Effect of DATS on bladder histopathology. Representative histological images of bladder sections from different treatment groups. Scale bar = 100 μm **(C)**. The thickness of bladder mucosa was analyzed as the percentage of control **(D)** (mean ± S.E.; n = 5; **P < 0.01). **(E–G)** Effect of DATS on CYP-induced bladder oxidative injury. The level of the bladder -SH groups and IgG was determined with a maleimide-labeling assay and Western blot analysis, respectively **(E)**. Densitometric analysis of the blots was shown in **(F,G)** and expressed as the percentage of control (mean analysis of the blots was shown in **(F,G)** and expressed as the percentage of control (mean ± S.E.; n = 3; *P < 0.05, **P < 0.01).

**FIGURE 7:**
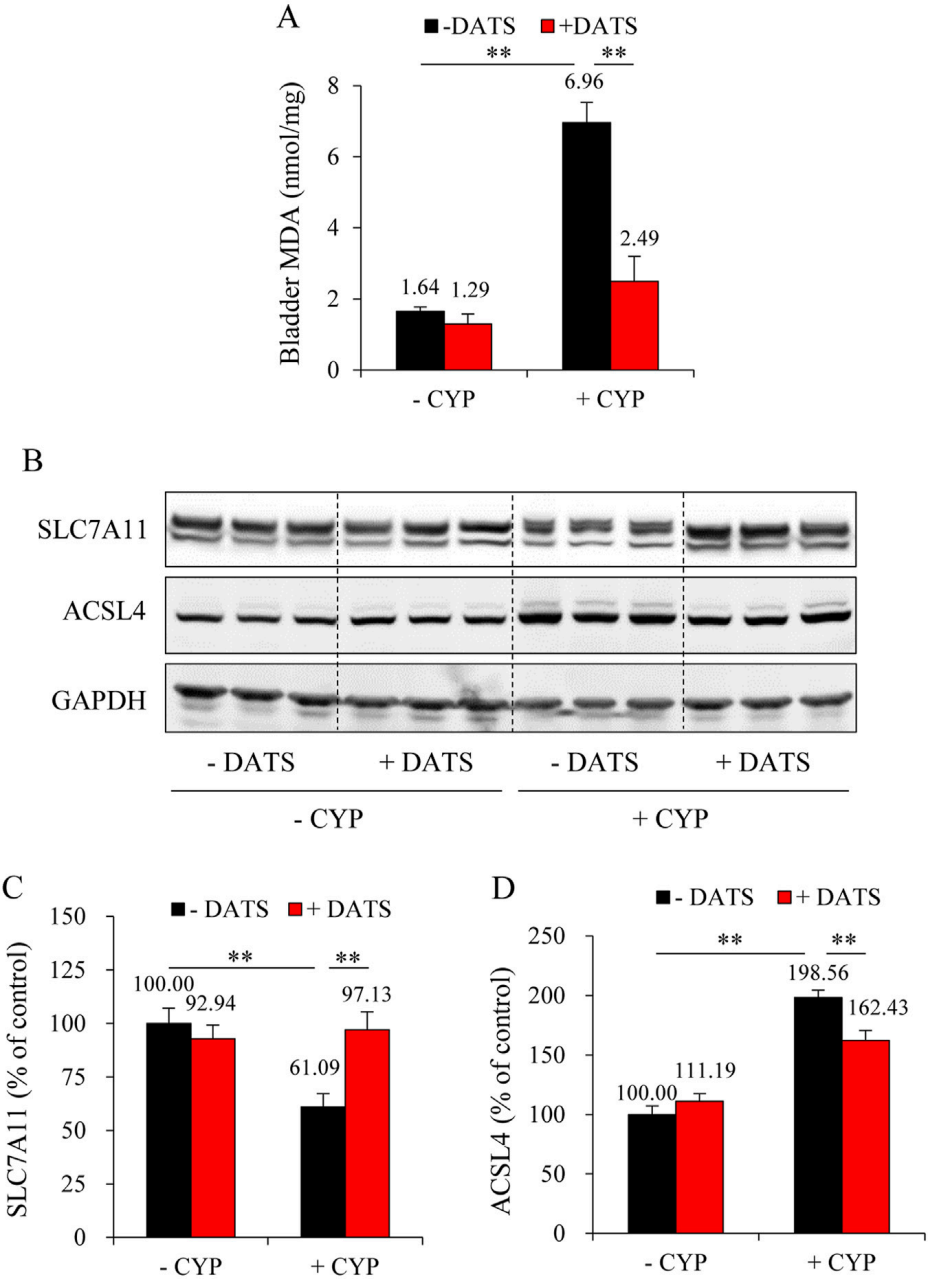
DATS prevents CYP-induced ferroptosis in the bladder. **(A)** Effect of DATS on bladder MDA production. The extracted bladder proteins were subjected to MDA assay following the manufacturer’s protocol (mean ± S.E.; n = 5; **P < 0.01). **(B–D)** Effect of DATS on ferroptosis markers in mouse bladder. Bladder proteins were analyzed with Western blot for SLC7A11, ACSL4, and GAPDH expression **(B)**. Densitometric analysis of the blots was shown in **(C,D)** and expressed as the percentage of control (mean ± S.E.; n = 3; **P < 0.01).

To further establish the role of H_2_S in mediating the protective effects of DR, we administered the H_2_S donor, DATS. Figure 6A shows that DATS treatment improved the macroscopic change in the bladder. It decreased the bladder-to-body weight ratio (Figure 6B). It also improved histochemical changes and preserved bladder mucosal thickness (Figures 6C,D). Additionally, DATS treatment markedly improved CYP-induced oxidative stress, inflammation, and ferroptotic changes in the bladder. Specifically, DATS mitigated the CYP-induced reduction in free sulfhydryl group (-SH) levels. It also prevented extravasation of CYP-induced immunoglobulin G (IgG) (Figures 6E–G). Furthermore, DATS reduced MDA levels (Figure 7A) and normalized the expression of ferroptosis markers SLC7A11 and ACSL4 (Figures 7B–D). These findings indicate that H_2_S plays a protective role in CYP-induced cystitis, and it mediates the beneficial effects of DR.

### 3.4 H_2_S protects urothelial cells from ACR-induced oxidative injury

**FIGURE 8:**
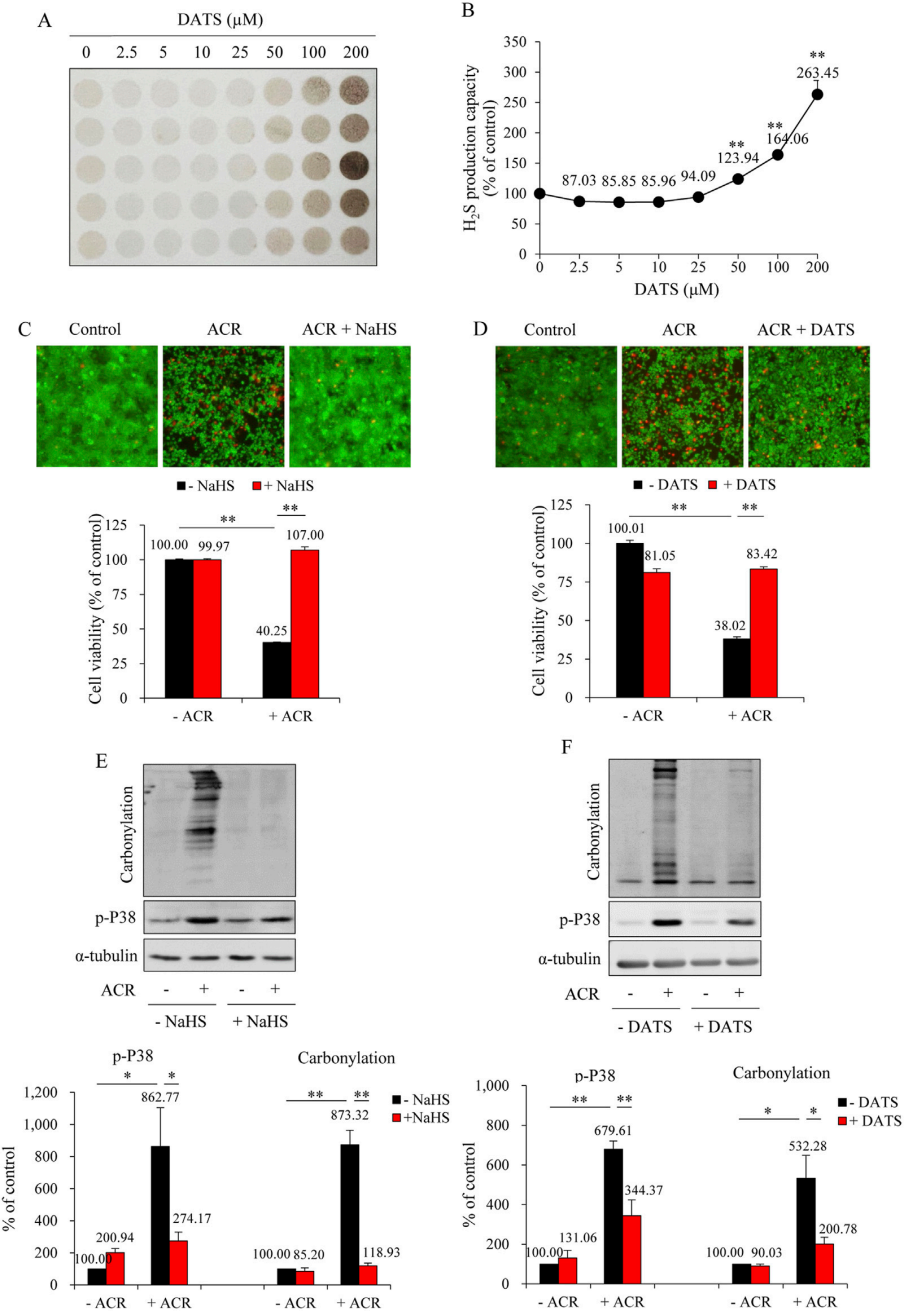
NaHS and DATS protect against ACR-induced urothelial cell injury. **(A,B)** Effect of DATS on H_2_S production capacity in urothelial cells. SV-HUC-1 cells treated with indicated concentrations of DATS were subjected to lead sulfide assay for 24 h **(A)**. Densitometric quantification of the intensity of the colored circles was performed, and data shown in **(B)** have been normalized to control (mean ± S.E.; n = 5; **P < 0.01). **(C,D)** Effect of NaHS and DATS on ACR-induced urothelial cell death. SV-HUC-1 cells were pretreated with 1 mM NaHS for 15 min or 25 μM DATS for 30 min before exposing to 100 μM ACR for an additional 24 h. The cell viability was determined by Calcein-AM/PI staining (upper) and WST assay (lower). Data shown are mean ± S.E. (n = 5 and 6, respectively; **P < 0.01). **(E,F)** Effect of NaHS and DATS on ACR-induced oxidative stress in urothelial cells. SV-HUC-1 cells were pretreated with 1 mM NaHS for 15 min or 25 μM DATS for 30 min before exposing to 75 μM ACR for an additional 4 h. Cell proteins were extracted for Western blot analysis of protein carbonylation and phosphorylated P38MAPK. A quantitative analysis of blots is shown below. Data are presented as mean ± S.E. (n = 3; *P < 0.05, **P < 0.01).

To further establish the protective role of H_2_S against ACR-induced bladder cell injury, we have used a cell culture model in which the role of H_2_S donors, DATS and NaHS, on ACR-elicited cell death and oxidative changes were analyzed. First, we confirmed the production of H_2_S when DATS was added into cultured urothelial cells. It caused a dose-dependent H_2_S generation (Figures 8A,B). Then, we analyzed the actions of NaHS and DATS on ACR-induced cell death. As shown in Figures 8C,D, NaHS and DATS similarly protected urothelial cells from ACR-induced cell death, as revealed by the reduced number of fluorescent red dead cells in Calcein-AM/PI staining and improved cell viability in WST assay. Consistently, NaHS and DATS potently prevented ACR-induced oxidative stress. The ACR-induced protein carbonylation and P38MAPK activation were markedly suppressed in the presence of NaHS and DATS (Figures 8E,F). These *in vitro* results provide direct evidence supporting a protective role of H_2_S on ACR-induced oxidative bladder cell injury.

## 4 Discussion

In this study, we demonstrated that DR protected mice against CYP-induced cystitis through mechanisms involving enhanced endogenous H_2_S production and inhibition of ferroptotic bladder injury. Our findings thus revealed a previously unrecognized link between DR, H_2_S signaling, and bladder protection. Given the multifaceted function of H_2_S and the broad implication of ferroptosis in various diseases, the finding provides novel mechanisms of the health benefit of DR. It suggests that DR could have therapeutic potential in a wide range of diseases.

In this study, we demonstrated that a short-term DR rendered mice resistant to CYP-induced cystitis, as evidenced by the reduced bladder weight, improved tissue damage, and decreased inflammatory and oxidative responses. It has long been recognized that ACR, the metabolite of CYP produced in the liver, mediates CYP toxicity in the bladder (Cox, 1979). The deposition of ACR in the urothelial cells initiates cell injury, subsequently activating inflammatory response, further worsening the situation. ACR, as a product of lipid peroxidation with a highly reactive nucleophilic group, elicits numerous toxic effects, primarily through induction of oxidative stress (Uchida, 1999; Uchida et al., 1998; Stevens and Maier, 2008). ACR stimulates reactive oxygen species (ROS) generation. It induces nicotinamide adenine dinucleotide phosphate (NADPH) oxidase expression in endothelial cells and macrophages, promoting superoxide generation (Kuntic et al., 2023). In addition, it depletes the major thiol antioxidant GSH and forms protein adducts with redox-sensitive proteins, thus influencing redox homeostasis (Stevens and Maier, 2008). As ROS is the primary factor initiating lipid peroxidation, the ROS induced by ACR triggers the production of more ACR, thus creating a vicious ACR-ROS-ACR cycle, propelling the progress of oxidative stress and cell damage (Moazamian et al., 2015; Liu-Snyder et al., 2006). In this study, we demonstrated that DR prevented CYP-induced lipid peroxidation and oxidative stress, as indicated by its inhibitory effects on ACR-protein adducts formation and MDA level, suggesting that DR may interrupt the vicious ACR-ROS-ACR cycle and prevented the progression of cell damage.

Ferroptosis, characterized by iron-dependent lipid peroxidation, has emerged as an essential mechanism in various pathological conditions (Liu et al., 2023; Martin-Sanchez et al., 2020; Xu et al., 2021; Yao et al., 2021; Zhang et al., 2022), including CYP-induced cystitis, as recently reported by our group (Mao et al., 2023a). There are also reports describing an implication of ferroptosis in CYP- and/or ACR-induced injuries in other organs, such as ovary, testis and neuron (Chen et al., 2024; Saleh et al., 2024; Zhang S. et al., 2023; Qi et al., 2022). Ferroptosis could be an important mechanism underlying CYP/ACR-related diseases.

In this study, we demonstrated that ferroptosis in CYP cystitis could potently prevented by DR. The questions naturally occurred as to how DR triggered body defense against CYP-initiated ferroptotic bladder injury. Our study revealed that elevated H_2_S generation could be critically involved. In support of this notion, we demonstrated that DR significantly elevated the H_2_S-synthesizing enzymes in the liver, which is known to be the significant source of H_2_S *in vivo* (Kamoun, 2004; Norris et al., 2011). In further support of the role of H_2_S, we have conducted loss-of-function and gain-of-function experiments. Inhibition of the major H_2_S-synthesizing enzymes CSE and CBS by their inhibitors PAG and AOAA abrogated the protective effects of DR on CYP-induced cystitis. In contrast, the supplement of H_2_S via administration of DATS, an H_2_S donor, mimicked the protective effects. These results demonstrated a mediating role of H_2_S in the observed effects of DR.

H_2_S has multiple biological functions. Our studies revealed that the protective effects of H_2_S are associated with significant suppression of oxidative stress and ferroptotic cell death pathways. H_2_S is known to be able to enhance cellular defense mechanisms against oxidative stress via the induction of endogenous antioxidative enzymes and GSH synthesis (Zhang et al., 2016; Yuan et al., 2017). It also has direct ROS-scavenging action (Pei et al., 2020). More recently, we and others have characterized H_2_S as a potent scavenger of lipid peroxidation products, ACR and 4-hydroxy-2-nonenal (4-HNE) (Mao et al., 2022; Laggner and Gmeiner, 2015; Schreier et al., 2010). In agreement with these previous observations, we observed that supplementing H_2_S with DATS and DR reduced lipid peroxidation product formation and protein oxidation in bladder tissue.

Furthermore, H_2_S suppressed ACR-induced protein carbonylation, P38MAPK activation, and oxidative cell death in cultured urothelial cells. Other than anti-oxidative actions, H_2_S has also been reported to be able to upregulate the expression of the molecules involved in cellular defense against ferroptosis, such as SLC7A11, GPX4 and ferritin (Zhang et al., 2024; Yu et al., 2023). In the current study, we also observed that DR tended to increase the basal level of SLC7A11.

In addition to mitigating oxidative stress and ferroptotic cell injury in the bladder, H_2_S exhibits potent anti-inflammatory properties, which likely contribute to its protective effects in CYP-induced cystitis. H_2_S is known to inhibit the activation of the NF-κB pathway and suppress inflammasome formation (Huang et al., 2016; Dilxat et al., 2024), thereby reducing the production of pro-inflammatory cytokines. Consistent with these anti-inflammatory actions, we observed that the H_2_S donor, DATS, significantly attenuated CYP-induced IgG extravasation, a marker of vascular inflammation and permeability. These findings suggest that the protective effects of DR are mediated, at least in part, by H_2_S-driven mechanisms, including its anti-oxidative, anti-inflammatory, and anti-ferroptotic actions.

Of note, our results show that DR upregulated H_2_S-synthesizing enzymes and elevated H_2_S production in the liver. The question naturally occurred as to how the increased H_2_S in the liver could exert distant effects on the bladder. One explanation is that the liver is also the primary organ for CYP metabolism and ACR formation (Marinello et al., 1984). The increased H_2_S in the liver could effectively scavenge locally produced ACR, thus providing the first line of defense against its toxicity. Moreover, as the predominant source of H_2_S *in vivo*, the liver-derived H_2_S in circulation could provide additional protection. There was one report showing that H_2_S production after DR was increased in liver and kidney, but not other organs (Bithi et al., 2021). Given that H_2_S-synthesizing enzymes have been documented in both rat and human bladders (Gai et al., 2013), a more detailed analysis of their expression under DR is warranted in the future to determine whether they contribute to the observed beneficial effects.

The mechanisms responsible for the elevation of H_2_S-synthesizing enzymes and subsequent H_2_S production under DR remain largely unclear. Studies by Hine et al. showed that the elevation could be due to the activation of transsulfuration pathway (TSP) because of sulfur amino acid (SAA) restriction (Hine et al., 2015). It could also be due to the reduced feedback regulation on liver H_2_S-producing enzymes by thyroid hormone (TH) and growth hormone (GH), both of which were known to be decreased following DR (Hine et al., 2017). These same events could also occur in the current study. More detailed investigations are needed to determine whether additional mechanisms could be involved.

Other than H_2_S, several alternative mechanisms have been proposed to explain the health benefits associated with DR. For example, DR activates autophagy (Bagherniya et al., 2018), which has been shown to be able to preserve mitochondrial function and reduce oxidative damage (Lee et al., 2012). DR also improves insulin sensitivity and mitigates systemic and skeletal muscle oxidative stress in type 2 diabetes (Rodrigues et al., 2011). Furthermore, DR has anti-inflammatory, immunomodulatory, and neuroendocrine adaptive effects (Cantoni et al., 2022). Moreover, DR promotes the expression of sirtuins, a family of enzymes that regulate numerous cellular processes (Cohen et al., 2004), including enhancing mitochondrial biogenesis and decreasing ROS generation (López-Lluch et al., 2006). The induction of mitochondrial SIRT3 by DR is reported to be associated with increased activity of antioxidant enzymes, such as superoxide dismutase 2 (SOD2) (Qiu et al., 2010; Someya et al., 2010). These pathways may collectively contribute to the health benefits observed with DR. Interestingly, many of the effects attributed to DR, such as improved insulin sensitivity and sirtuin induction, could also be similarly achieved by H_2_S (Bełtowski et al., 2018; Xin et al., 2016). Thus, it is likely that these mechanisms are interconnected and reciprocal regulated. In addition to H_2_S, other pathways may also play a role in the protective effects observed in this study.

Our study could have significant implications. Although the beneficial effects of DR have been documented in several animal models, our study is the first to demonstrate such protection in a chemical-induced cystitis model. Notably, our findings indicate that even 1-week period of DR can significantly enhance tissue resistance to injury, suggesting that short-term dietary modifications may have profound effects on health. Furthermore, our study characterized H_2_S, produced by the liver, as a potential mechanism underlying the protective actions of DR, providing a mechanistic link between systemic metabolic adaptation and organ-specific protection. Additionally, our research offers novel insights into the mechanisms of CYP-induced cystitis. We confirmed the critical involvement of ferroptosis in bladder injury and demonstrated that H_2_S donors could serve as new therapeutic agents for treating CYP-induced cystitis. It is especially worth mentioning that DATS, as a natural compound found in garlic with a good safety profile, could be a good candidate for therapeutic purposes. Lastly, since oxidative stress and ferroptosis have been implicated in a wide range of diseases, the ability of DR to modulate these pathways via H_2_S suggests that similar strategies may be effective in conditions such as inflammatory diseases, ischemia-reperfusion injury, and other forms of chemical-induced tissue damage.

Of note, although this study provides a novel concept about the potential use of DR and H_2_S in the prevention of chemical-induced bladder cystitis, it is still an early stage of concept. More detailed studies are needed to establish clinical relevance. Moreover, this study also has several limitations. First, while we demonstrated the protective effects of DR and H_2_S in a CYP-induced cystitis model, it remains unclear whether these findings can be generalized to other forms of bladder injury or to other organs. Second, in this study, DR was applied for only 1 week. It is unclear whether the long-term DR regimens could achieve persistent, or even greater protective effects. Third, although DR is associated with numerous benefits, long-term or extreme food restriction may lead to adverse effects, such as impaired immunity, malnutrition, and metabolic imbalances (Jolly, 2007; Dung et al., 2023). Therefore, the duration and intensity of DR, as well as the individual circumstances of patients, should be carefully evaluated when considering DR as a therapeutic approach. Fourth, while we identified H_2_S as a key mediator of protection, the potential role of other mechanisms, such as autophagy and mitochondrial adaptation, warrants further investigation. Additionally, the effects of H_2_S on inflammation and cell death are highly context-dependent, with both protective and exacerbating actions observed depending on its concentration and physiological context (Hegde and Bhatia, 2011; Yang, 2011). Careful consideration of H_2_S levels and their potential effects is needed. Finally, the feasibility of using garlic-derived compounds in clinical settings should be further explored to evaluate their safety, efficacy, and practical application.

In conclusion, our study demonstrates that DR protects against CYP-induced cystitis through enhancing H_2_S production and subsequent modulation of oxidative stress and ferroptotic pathways. These findings provide new insights into the mechanisms by which DR enhances tissue protection and suggest novel strategies for preventing chemical-induced bladder injury. Given the importance of H_2_S in body homeostasis and the widespread role of oxidative stress and ferroptosis in disease pathogenesis, our findings may have broader implications for understanding the beneficial effects of DR and its practical application in various pathological conditions.

## Data Availability

The raw data supporting the conclusions of this article will be made available by the authors, without undue reservation.
